# Perception, knowledge and attitudes of Ecuadorian dentists towards patients with molar incisor hypomineralization

**DOI:** 10.3389/froh.2026.1789741

**Published:** 2026-06-08

**Authors:** Ana Armas-Vega, Juan Marcos Parise-Vasco, Jaime Angamarca-Iguago, Jaen Carlos Cagua-Ordoñez, Claudia Reytor-González, Jennifer Ribadeneira-Morales, Cassiano K. Rösing, Fernanda Noal, Daniel Simancas-Racines

**Affiliations:** 1 Facultad de Ciencias de la Salud, Universidad de Los Hemisferios, Quito, Ecuador; 2 Facultad de Odontología, Universidad Central del Ecuador, Quito, Ecuador; 3 Facultad de Ciencias de la Salud y Bienestar Humano, Universidad Tecnológica Indoamérica, Quito, Ecuador; 4 Facultad de Ciencias de la Salud y Bienestar Humano, Universidad Tecnológica Indoamérica, Ambato, Ecuador; 5 Facultad de Salud y Bienestar, Pontificia Universidad Católica del Ecuador, Quito, Ecuador; 6 Universidade Federal do Rio Grande do Sul, Porto Alegre, Brazil; 7 Universidad Científica del Sur, Lima, Peru

**Keywords:** attitude, dentistry, knowledge, molar incisor hypomineralization, perception, continuing education, health workforce

## Abstract

**Background:**

Molar Incisor Hypomineralization (MIH) is one of the most prevalent enamel development defects in contemporary dental practice. Understanding perceptions, knowledge, and attitudes of professionals towards this condition is key to optimizing clinical protocols and improving therapeutic results.

**Objective:**

To evaluate the perception, knowledge and attitudes of Ecuadorian dentists about diagnosis and management of patients with MIH.

**Methods:**

A cross-sectional observational study was carried out using a self-administered survey of registered dentists in Ecuador. A validated 19-question questionnaire was applied that explored demographic characteristics, knowledge of etiology and prevalence, attitudes towards clinical management, and therapeutic choices. In addition, two clinical cases with photographic support were included to evaluate treatment decisions. The statistical analysis incorporated descriptive statistics, chi-square or Fisher's exact tests, and multivariate logistic regression models.

**Results:**

A total of 352 dentists participated, predominantly working in private practice (71.9%), particularly in general dentistry (61.1%). Most practitioners reported observing MIH monthly (45.7%) and perceived an increase in its occurrence (67.0%). Demarcated opacities were the most frequent clinical manifestation (93.2%), with antibiotics being the main reported etiological factor (31.8%). The frequency of observation varied significantly between specialties (*p* < 0.05). Management was considered difficult, with durability of restorations being the main concern. Glass ionomer (37.5–71.4%) was the primary therapeutic choice, followed by fluoride varnish (22.6–37.5%). In the multivariate logistic regression model, professionals with 6–10 years of experience showed significantly higher odds of adequate knowledge (adjusted OR = 2.23, 95% CI: 1.14–4.37, *p* = 0.019). No independent predictors of favorable attitude towards MIH management reached statistical significance after multivariate adjustment.

**Conclusions:**

MIH is recognized as a growing and complex problem. It is frequently observed with demarcated opacities as the predominant sign and antibiotics as the main etiological factor. Its management is challenging, and professional experience appears to influence clinical knowledge. Continuing education and the development of standardized clinical protocols are needed to strengthen professional competence in MIH management.

## 1 Introduction

The term Molar Incisor Hypomineralization (MIH) was introduced in the beginning of the 21st century (1), and is related to a qualitative defect in the development of tooth enamel, not related to the presence of microorganisms (1, 2). In this alteration, produced during amelogenesis, the ameloblasts, in their maturation phase, undergo modifications, with excessive expansion of the crystals in length and thickness. These characteristics are visualized in changes in thickness and volume that affect the hardness of the enamel in an intensity dependent on the stage of development, extension and duration, in which the alteration occurs (1, 3–5).

Its etiology, little clarified so far, is associated with diseases of the mother during pregnancy, consumption of certain drugs during the gestation period, food contamination, neonatal alterations, pathologies in early childhood, and genetic factors that are yet to be clarified (6–8). The clinical characteristics of MIH vary according to severity, from alterations in the form of opaque spots on the tip of the cusp of the first and second molars or on free surfaces of the incisors and permanent upper or lower molars (6), to macroscopic loss of enamel proportional to the increase in porosity (9).

MIH is considered to be one of the pathologies that has been increasingly understood and therefore detected more frequently (10). Its reported prevalence in countries of the Americas is between 20% and 29% (4). The limited knowledge of the pathology among dentists and the masking of the lesion, due to its association with carious lesions secondary to its presence, constitute the greatest difficulty in the diagnosis of pathology (11). The signs of MIH on the tooth surface trigger biofilm retention, development of carious lesions, gum disease and even tooth loss (4), cases of pain, anxiety, alterations in self-esteem and confidence in the sufferer (12). Early diagnosis of MIH ensures early intervention and conservative treatments with better results (13).

Several instruments have been used to assess dentists’ knowledge and attitudes towards MIH, including a questionnaire validated by Serna-Muñoz (14), and subsequently applied in multiple countries including Greece, Norway, Hong Kong, Syria, Switzerland, France, and Turkey (9, 13, 15–20). However, evidence from Latin America remains lacking. In Ecuador, data on MIH prevalence, professional preparedness, and standardized approaches to diagnosis and management are limited (21–23). This gap is notable given the reported burden of developmental enamel defects, the unequal distribution of the dental workforce, and the absence of a local evidence base to inform education, clinical guidance, and public health action. The aim of this study is to evaluate the perceptions, knowledge and attitudes of dentists who are members of the Ecuadorian Dental Federation (FOE) towards MIH, using a previously validated instrument.

## 2 Methods

An analytical cross-sectional observational study was carried out between October and December 2023, after obtaining approval from the Research Ethics Committee on Human Subjects of the Central University of Ecuador (CEISH-UCE), in ordinary session No. 035-CEISH-UCE-2023, dated October 10, 2023 (protocol code: 011-DOC-FO-2023). The target population consisted of practicing dentists affiliated with the Ecuadorian Dental Federation, a national professional association open to all dentists, including recent graduates, regardless of their level of education, with a total universe of 3,880 registered professionals at the time of the study. The sample size was determined based on convenience and the finite population. A 95% confidence level and a maximum acceptable margin of error of 5% were used, as well as an assumed expected prevalence of 50% for the main variables of interest. The calculation resulted in a minimum required sample of 359 participants, selected through non-probabilistic convenience sampling.

Inclusion criteria included dentists with a valid university degree recognized in Ecuador, active professional practice at the time of the study, current affiliation to the Ecuador Dental Federation, and voluntary acceptance of participation through electronic informed consent. Dental students or rotating interns, retired professionals or professionals without active clinical practice, incomplete questionnaires with more than 20% of missing responses, and responses with inconsistent patterns suggesting non-genuine completion were not included.

During the break in five FOE members’ meetings to address professional matters in different cities throughout the country, the principal investigator presented the project, including its potential benefits, associated risks, objectives, and proposed methodology. After identifying members who expressed interest in participating, the voluntary nature of their participation was emphasized. The professionals who agreed to participate provided their informed consent by signing a physical document, as required by the CEISH-UCE, before accessing the questionnaire. Once this step was completed and the participation was confirmed, each participant received a unique link by telephone, generated through Google Forms, requesting them to complete the questionnaire. This link led to a self-administered survey developed from a previously validated instrument. The average completion time was 8–12 min, and the principal investigator was present with each participant during the meeting to address any difficulties that might arise during the survey process. An automated reminder system was implemented 7 and 14 days after the initial submission to optimize the response rate.

The convenience sampling approach, limited to attendees of FOE meetings, may have introduced selection bias by excluding non-attending professionals. Additionally, the voluntary nature of participation may have favored enrollment of those with greater interest in MIH, leading to possible volunteer bias. To mitigate these limitations, recruitment was conducted across five cities throughout the country to ensure geographic diversity, and an automated reminder system was implemented to maximize response rates.

The questionnaire was obtained from the original author (14), who provided the instrument in the language in which it was originally administered (European Spanish). Consequently, a cross-cultural adaptation validation was carried out by presenting the questionnaire to a focus group of five pediatric dentistry specialists, who evaluated the clarity and comprehensibility of the terms used. No modifications were deemed necessary. Consequently, the questionnaire was structured into three main sections designed to comprehensively assess knowledge, attitudes, and practices related to molar-incisor hypomineralization. The first section evaluated sociodemographic and professional characteristics using eight items, including age, sex, years of professional experience, province of practice, type of residence, sector of work, academic level and area of clinical specialization. The second section explored knowledge and attitudes towards molar incisor hypomineralization through multiple-choice items (14), evaluating perception of frequency and prevalence, most observed clinical manifestations, identified etiological factors, perceptions about the complexity of clinical management, main difficulties encountered in treatment, sources of information consulted, and additional training needs. The third section presented two clinical cases with detailed textual descriptions and high-quality clinical images provided by the questionnaire’s author (14). These cases were included to evaluate therapeutic decision-making in realistic clinical scenarios. The first case corresponded to a 7-year-old patient with a semi-erupted permanent first molar presenting moderate molar incisor hypomineralization, post-eruptive fracture and dental sensitivity, offering as therapeutic options application of fluoride varnish, restoration with glass ionomer, restoration with composite resin, tooth extraction, and the alternative “I am not sure”. The second case presented a delimited lesion with brown opacity without post-eruptive enamel fracture, structuring the options by combining tissue removal strategies with specific restorative materials. The questionnaire was used with the written authorization of the corresponding author of the original instrument (14). The complete questionnaire and the clinical images used in both cases are available in the original publication (14).

The statistical analysis was performed using R v4.5.1 software. Descriptive analysis included absolute frequencies and percentages for categorical variables and means with standard deviations for continuous variables. The normality of the continuous variables was assessed using the Kolmogorov–Smirnov test and graphical analysis.

Two binary dependent variables were constructed based on clinical criteria established in the scientific literature: (a) “adequate knowledge about MIH,” defined as the correct identification of demarcated opacities as the main clinical manifestation combined with the recognition of evidence-based etiological factors (antibiotics and medications, or genetic factors); and (b) “favorable attitude towards MIH management,” defined as not perceiving treatment as extremely difficult, indicating appropriate clinical confidence for the therapeutic approach.

Bivariate analysis evaluated associations between demographic, occupational, and the dependent variables using Pearson’s chi-square test for categorical variables, reporting exact two-tailed *p*-values. When expected cell frequencies were below 5, Fisher’s exact test was applied. Crude odds ratios with 95% confidence intervals were calculated for statistically significant or clinically relevant associations.

Multivariate logistic regression models were developed to identify independent predictors of the two dependent variables, including as covariates: dental specialty, professional experience, academic level, work sector, age group, sex, and previous information on MIH. Goodness of fit was assessed using the Hosmer-Lemeshow test and Nagelkerke’s pseudo-R2. The discriminative ability of each model was evaluated using the area under the receiver operating characteristic curve (AUC-ROC). Results are presented as adjusted odds ratios with 95% confidence intervals. A significance level of *α*=0.05 was established for all tests, handling missing data through complete case analysis.

The results were reported following STROBE guidelines for cross-sectional observational studies (24). The study was carried out following the ethical principles established in the Declaration of Helsinki, guaranteeing confidentiality and anonymity of the data through numerical coding without personal identifiers. Participation was voluntary with the possibility of withdrawal at any time without consequences, storing the collected data on secure servers with access restricted exclusively to the research team.

## 3 Results

### 3.1 Sociodemographic characteristics

The final sample comprised 352 Ecuadorian dentists, with a predominantly female distribution (70.2%, *n* = 247). The most represented age group was 31–40 years (42.3%, *n* = 149), followed by professionals aged 30 years or younger (31.8%, *n* = 112). Most respondents resided in urban areas (90.9%, *n* = 320), with a smaller proportion in rural settings (9.1%, *n* = 32). Participants were geographically distributed across 22 of Ecuador’s 24 provinces, with the largest proportion from Pichincha (41.2%, *n* = 145), followed by Guayas (9.7%, *n* = 34), Manabí (7.7%, *n* = 27), Imbabura (6.8%, *n* = 24), and Chimborazo (6.5%, *n* = 23). No participants were recorded from Bolivar or Zamora Chinchipe.

In terms of professional experience, 41.2% (*n* = 145) reported five years or less of clinical practice, while 22.7% (*n* = 80) had more than 15 years of experience. The distribution by labor sector showed a marked predominance of private practice (71.9%, *n* = 253), with a lower representation of the public sector (16.2%, *n* = 57) and mixed practice (11.9%, *n* = 42). Regarding academic level, 59.1% (*n* = 208) held a third-level degree (undergraduate), 27.8% (*n* = 98) had completed a specialization, 9.9% (*n* = 35) had a master’s degree, and 3.1% (*n* = 11) held a doctoral degree. Most participants (61.1%, *n* = 215) practiced general dentistry, followed by pediatric dentistry (8.0%, *n* = 28). Table 1 summarizes the sociodemographic characteristics of the participants according to dental specialties.

**Table 1:** Sociodemographic characteristics of the participants considering specialties.

Sociodemographic variable	Total *n* = 352	General dentistry *n* = 215	Pediatric dentistry *n* = 28	Orthodontics *n* = 24	Endodontics *n* = 23	Oral Rehabilitation *n* = 19	Dental Aesthetics *n* = 12	Other specialties^a^ *n* = 31
Sex								
Female (%) Male (%)	247 (70.2) 105 (29.8)	153 (71.2) 62 (28.8)	25 (89.3) 3 (10.7)	14 (58.3) 10 (41.7)	15 (65.2) 8 (34.8)	11 (57.9) 8 (42.1)	10 (83.3) 2 (16.7)	19 (61.3) 12 (38.7)
Age								
≤ 30 years (%) 31–40 years (%) 41–50 years (%) > 50 years (%)	112 (31.8) 149 (42.3) 43 (12.2) 48 (13.6)	94 (43.7) 82 (38.1) 16 (7.4) 23 (10.7)	4 (14.3) 12 (42.9) 5 (17.9) 7 (25.0)	5 (20.8) 10 (41.7) 7 (29.2) 2 (8.3)	6 (26.1) 10 (43.5) 4 (17.4) 3 (13.0)	1 (5.3) 9 (47.4) 3 (15.8) 6 (31.6)	0 (0.0) 8 (66.7) 3 (25.0) 1 (8.3)	2 (6.5) 18 (58.1) 5 (16.1) 6 (19.4)
Years of practice								
≤ 5 years (%) 6–10 years 11–15 years > 15 years	145 (41.2) 88 (25.0) 39 (11.1) 80 (22.7)	112 (52.1) 52 (24.2) 16 (7.4) 35 (16.3)	8 (28.6) 4 (14.3) 3 (10.7) 13 (46.4)	5 (20.8) 6 (25.0) 7 (29.2) 6 (25.0)	9 (39.1) 5 (21.7) 3 (13.0) 6 (26.1)	3 (15.8) 8 (42.1) 3 (15.8) 5 (26.3)	1 (8.3) 5 (41.7) 2 (16.7) 4 (33.3)	7 (22.6) 8 (25.8) 5 (16.1) 11 (35.5)
Work sector								
Public Private Both	57 (16.2) 253 (71.9) 42 (11.9)	35 (16.3) 165 (76.7) 15 (7.0)	6 (21.4) 19 (67.9) 3 (10.7)	3 (12.5) 19 (79.2) 2 (8.3)	4 (17.4) 16 (69.6) 3 (13.0)	2 (10.5) 14 (73.7) 3 (15.8)	1 (8.3) 8 (66.7) 3 (25.0)	6 (19.4) 12 (38.7) 13 (41.9)
Academic degree								
Third level Specialization Master Phd	208 (59.1) 98 (27.8) 35 (9.9) 11 (3.1)	207 (96.3) 1 (0.5) 6 (2.8) 1 (0.5)	0 (0.0) 19 (67.9) 5 (17.9) 4 (14.3)	1 (4.2) 18 (75.0) 3 (12.5) 2 (8.3)	0 (0.0) 17 (73.9) 6 (26.1) 0 (0.0)	0 (0.0) 16 (84.2) 3 (15.8) 0 (0.0)	0 (0.0) 10 (83.3) 2 (16.7) 0 (0.0)	0 (0.0) 17 (54.8) 10 (32.3) 4 (12.9)

a Other specialties include Preventive Dentistry, Hospital Management, Periodontics, Public Health, Maxillofacial Surgery, Oral Surgery, Implantology, Pathology, Epidemiology.

### 3.2 Perception of MIH prevalence and clinical features

Most dentists (45.7%, *n* = 161) observed cases of MIH monthly, while 33.5% (*n* = 118) identified them annually and 20.7% (*n* = 73) weekly. Regarding estimated prevalence, 58.5% (*n* = 206) considered that less than 10% of their patients had MIH, 34.9% (*n* = 123) estimated a prevalence of 10%–25%, and 6.5% (*n* = 23) considered a prevalence above 25%. Additionally, 67.0% (*n* = 236) perceived an increase in MIH incidence in recent years. Most participants (93.2%, *n* = 328) correctly identified demarcated opacities as the most frequently observed clinical manifestation, while 51.1% (*n* = 180) considered that MIH occurs less frequently in second primary molars compared to permanent first molars.

The frequency of observation of hypomineralized teeth varied significantly between specialties (*p* < 0.05, Fisher’s exact test); however, given the small size of some subgroups, these results should be interpreted with caution. The estimated prevalence of patients with MIH also differed significantly between specialties (*p* < 0.05). The perception of increased incidence, clinical characteristics observed, and frequency in second primary molars did not show significant differences between specialties (*p* > 0.05 for all) (Table 2).

**Table 2:** Frequency of the level of perception of molar incisor hypomineralization among the participants.

Perception variable	Total *n* = 352	General dentistry *n* = 215	Pediatric dentistry *n* = 28	Orthodontics *n* = 24	Endodontics *n* = 23	Oral Rehabilitation *n* = 19	Dental Aesthetics *n* = 12	Other specialties^a^ *n* = 31	*p*-value
Frequency of Hypomineralized Teeth									
Weekly (%) Monthly (%) Annually (%)	73 (20.7) 161 (45.7) 118 (33.5)	42 (19.5) 98 (45.6) 75 (34.9)	9 (32.1) 16 (57.1) 3 (10.7)	4 (16.7) 14 (58.3) 6 (25.0)	3 (13.0) 8 (34.8) 12 (52.2)	6 (31.6) 9 (47.4) 4 (21.1)	4 (33.3) 6 (50.0) 2 (16.7)	5 (16.1) 10 (32.3) 16 (51.6)	0.052^b^
Percentage of patients who have this defect									
Lower 10% (%) 10% to 25% (%) More than 25% (%)	206 (58.5) 123 (34.9) 23 (6.5)	138 (64.2) 62 (28.8) 15 (7.0)	10 (35.7) 16 (57.1) 2 (7.1)	10 (41.7) 10 (41.7) 4 (16.7)	13 (56.5) 9 (39.1) 1 (4.3)	11 (57.9) 8 (42.1) 0 (0.0)	2 (16.7) 10 (83.3) 0 (0.0)	22 (71.0) 8 (25.8) 1 (3.2)	0.002^b^
Incidence of MIH and its increase in recent years									
Yes (%) No (%)	236 (67.0) 116 (33.0)	136 (63.3) 79 (36.7)	25 (89.3) 3 (10.7)	18 (75.0) 6 (25.0)	14 (60.9) 9 (39.1)	12 (63.2) 7 (36.8)	10 (83.3) 2 (16.7)	21 (67.7) 10 (32.3)	0.112^c^
Characteristics you notice in patients									
Demarcated opacities Post-eruption enamel breakdown	328 (93.2) 24 (6.8)	205 (95.3) 10 (4.7)	25 (89.3) 3 (10.7)	22 (91.7) 2 (8.3)	20 (87.0) 3 (13.0)	17 (89.5) 2 (10.5)	11 (91.7) 1 (8.3)	28 (90.3) 3 (9.7)	0.601^b^
Frequency of this problem in the second temporal molar									
Just as often Less often More often	108 (30.7) 180 (51.1) 64 (18.2)	67 (31.2) 111 (51.6) 37 (17.2)	9 (32.1) 12 (42.9) 7 (25.0)	6 (25.0) 15 (62.5) 3 (12.5)	6 (26.1) 14 (60.9) 3 (13.0)	6 (31.6) 6 (31.6) 7 (36.8)	5 (41.7) 4 (33.3) 3 (25.0)	9 (29.0) 18 (58.1) 4 (12.9)	0.549^b^

a Other specialties include Preventive Dentistry, Hospital Management, Periodontics, Public Health, Maxillofacial Surgery, Oral Surgery, Implantology, Pathology, Epidemiology.

b Fisher’s exact test (Monte Carlo simulation) used when >20% of cells had expected frequencies <5. *P*-values were calculated to assess differences between dental specialties. Due to small subgroup sizes in some specialties, these comparisons should be considered exploratory.

c *P*-value were calculated using Pearson’s chi-square test.

### 3.3 Attitudes towards clinical management and therapeutic decisions

Most professionals considered MIH management somewhat or very difficult, with the perception of management difficulty not differing significantly between specialties (*p* > 0.05). The main difficulties identified varied significantly between specialties (*p* < 0.05), with long-term restoration success as the predominant concern across all groups. Sources of information and additional training needs did not differ significantly between specialties (*p* > 0.05). The Internet was the most frequently consulted source, and comprehensive training covering all aspects was the predominant need (>60% in all specialties) (Table 3).

**Table 3:** Perceptions of MIH by dental specialty.

Attitudinal variable	Total *n* = 352	General dentistry *n* = 215	Pediatric dentistry *n* = 28	Orthodontics *n* = 24	Endodontics *n* = 23	Oral Rehabilitation *n* = 19	Dental Aesthetics *n* = 12	Other specialties^a^ *n* = 31	*p*-value
Management of MIH as a clinical challenge									
Not a challenge (%) Yes, somewhat difficult (%) Yes, very difficult (%)	41 (11.6) 221 (62.8) 90 (25.6)	26 (12.1) 139 (64.7) 50 (23.3)	4 (14.3) 15 (53.6) 9 (32.1)	3 (12.5) 13 (54.2) 8 (33.3)	3 (13.0) 14 (60.9) 6 (26.1)	0 (0.0) 17 (89.5) 2 (10.5)	1 (8.3) 6 (50.0) 5 (41.7)	4 (12.9) 17 (54.8) 10 (32.3)	0.521^b^
Main difficulties									
Diagnosis (%) Aesthetics (%) Long-term success in restoration Provide Successful Restore Correct Margin Determination Obtaining correct local anaesthesia	75 (21.3) 51 (14.5) 156 (44.3) 39 (11.1) 27 (7.7) 4 (1.1)	49 (22.8) 32 (14.9) 95 (44.2) 25 (11.6) 12 (5.6) 2 (0.9)	2 (7.1) 3 (10.7) 17 (60.7) 2 (7.1) 3 (10.7) 1 (3.6)	3 (12.5) 3 (12.5) 13 (54.2) 3 (12.5) 2 (8.3) 0 (0.0)	9 (39.1) 5 (21.7) 3 (13.0) 2 (8.7) 3 (13.0) 1 (4.3)	3 (15.8) 2 (10.5) 7 (36.8) 5 (26.3) 2 (10.5) 0 (0.0)	3 (25.0) 2 (16.7) 6 (50.0) 0 (0.0) 1 (8.3) 0 (0.0)	6 (19.4) 4 (12.9) 15 (48.4) 2 (6.5) 4 (12.9) 0 (0.0)	0.378^b^
Where you get information									
Continuing Education (%) Internet (%) Books (%) Magazines (%)	84 (23.9) 205 (58.2) 32 (9.1) 31 (8.8)	50 (23.3) 131 (60.9) 17 (7.9) 17 (7.9)	10 (35.7) 9 (32.1) 5 (17.9) 4 (14.3)	3 (12.5) 18 (75.0) 1 (4.2) 2 (8.3)	9 (39.1) 10 (43.5) 1 (4.3) 3 (13.0)	4 (21.1) 13 (68.4) 1 (5.3) 1 (5.3)	4 (33.3) 4 (33.3) 3 (25.0) 1 (8.3)	4 (12.9) 20 (64.5) 4 (12.9) 3 (9.7)	0.129^b^
Needs for additional information									
Diagnosis (%) Etiology (%) Treatment (%) All of the above (%)	35 (9.9) 29 (8.2) 56 (15.9) 232(65.9)	23 (10.7) 19 (8.8) 33 (15.3) 140 (65.1)	0 (0.0) 2 (7.1) 8 (28.6) 18 (64.3)	3 (12.5) 3 (12.5) 3 (12.5) 15 (62.5)	2 (8.7) 3 (13.0) 1 (4.3) 17 (73.9)	4 (21.1) 0 (0.0) 0 (0.0) 15 (78.9)	0 (0.0) 0 (0.0) 2 (16.7) 10 (83.3)	3 (9.7) 2 (6.5) 9 (29.0) 17 (54.8)	0.158^b^

a Other specialties include Preventive Dentistry, Hospital Management, Periodontics, Public Health, Maxillofacial Surgery, Oral Surgery, Implantology, Pathology, Epidemiology.

b *P*-values were calculated using Fisher's exact test with Monte Carlo simulation, given that >20% of cells had expected frequencies <5. Due to small subgroup sizes in some specialties, these comparisons should be considered exploratory.

In relation to etiological factors, participants were asked to identify the single most relevant factor. Antibiotics and medications were selected by 31.8% (*n* = 112), followed by genetic factors (21.9%, *n* = 77) and acute medical conditions (14.8%, *n* = 52). The identification of causal factors showed homogeneous patterns between specialties (*p* > 0.05).

Therapeutic decisions for both clinical cases did not differ significantly between specialties (*p* > 0.05). Glass ionomer restoration was the most selected option for Case 1 across all groups (range: 37.5–71.4%), with fluoride varnish as the second most frequent choice (range: 22.6–37.5%). For Case 2 (delimited brown opacity), conservative strategies predominated, with greater variability in therapeutic options reflecting the diversity of clinical approaches available (Table 4).

**Table 4:** Level of knowledge and therapeutic decisions.

Knowledge and therapeutic-decision variable	Total *n* = 352	General dentistry *n* = 215	Pediatric dentistry *n* = 28	Orthodontics *n* = 24	Endodontics *n* = 23	Oral Rehabilitation *n* = 19	Dental Aesthetics *n* = 12	Other specialties^a^ *n* = 31	*p*-value
Factors involved in the etiology of MIH									
Antibiotics/medication (%) Acute medical condition (%) Chronic medical condition (%) Environmental pollutants (%) Fluoride exposure (%) Genetic factors (%)	112 (31.8) 52 (14.8) 28 (8.0) 43 (12.2) 40 (11.4) 77 (21.9)	68 (31.6) 27 (12.6) 21 (9.8) 28 (13.0) 24 (11.2) 47 (21.9)	9 (32.1) 4 (14.3) 4 (14.3) 3 (10.7) 0 (0.0) 8 (28.6)	10 (41.7) 2 (8.3) 0 (0.0) 5 (20.8) 3 (12.5) 4 (16.7)	8 (34.8) 5 (21.7) 0 (0.0) 2 (8.7)4 (17.4) 4 (17.4)	7 (36.8) 4 (21.1) 0 (0.0) 2 (10.5) 1 (5.3) 5 (26.3)	2 (16.7) 4 (33.3) 0 (0.0) 0 (0.0) 3 (25.0) 3 (25.0)	8 (25.8) 6 (19.4) 3 (9.7) 3 (9.7) 5 (16.1) 6 (19.4)	0.538^b^
Case 1: Temporal molar with moderate MIH									
Fluoride varnish (%) Ionomer restoration (%) Resin restoration (%) Tooth extraction (%) Not sure (%)	115 (32.7) 167 (47.4) 24 (6.8) 1 (0.3) 45 (12.8)	76 (35.3) 95 (44.2) 13 (6.0) 1 (0.5) 30 (14.0)	8 (28.6) 20 (71.4) 0 (0.0) 0 (0.0) 0 (0.0)	9 (37.5) 9 (37.5) 3 (12.5) 0 (0.0) 3 (12.5)	7 (30.4) 10 (43.5) 1 (4.3) 0 (0.0) 5 (21.7)	5 (26.3) 9 (47.4) 2 (10.5) 0 (0.0) 3 (15.8)	3 (25.0) 8 (66.7) 0 (0.0) 0 (0.0) 1 (8.3)	7 (22.6) 16 (51.6) 5 (16.1) 0 (0.0) 3 (9.7)	0.509^b^
Case 2: Delimited brown opacity									
Do not remove tissue + ionomer (%) Remove affected tissue + ionomer Remove affected tissue + resin (%) Remove all tissue + resin (%) Remove all tissue + ionomer (%) Do not remove tissue + resin (%) Other options (%)	80 (22.7) 77 (21.9) 42 (11.9) 53 (15.1) 45 (12.8) 36 (10.2) 19 (5.4)	48 (22.3) 41 (19.1) 22 (10.2) 36 (16.7) 30 (14.0) 26 (12.1) 12 (5.6)	8 (28.6) 10 (35.7) 4 (14.3) 1 (3.6) 4 (14.3) 1 (3.6) 0 (0.0)	4 (16.7) 5 (20.8) 3 (12.5) 4 (16.7) 4 (16.7) 2 (8.3) 2 (8.3)	4 (17.4) 6 (26.1) 3 (13.0) 5 (21.7) 2 (8.7) 0 (0.0) 3 (13.0)	5 (26.3) 4 (21.1) 4 (21.1) 3 (15.8) 1 (5.3) 1 (5.3) 1 (5.3)	4 (33.3) 3 (25.0) 1 (8.3) 1 (8.3) 0 (0.0) 3 (25.0) 0 (0.0)	7 (22.6) 8 (25.8) 5 (16.1) 3 (9.7) 4 (12.9) 3 (9.7) 1 (3.2))	0.814^b^

a Other specialties include Preventive Dentistry, Hospital Management, Periodontics, Public Health, Maxillofacial Surgery, Oral Surgery, Implantology, Pathology, Epidemiology.

b *P*-values were calculated using Fisher's exact test with Monte Carlo simulation, given that >20% of cells had expected frequencies <5. Due to small subgroup sizes in some specialties, these comparisons should be considered exploratory.

### 3.4 Predictors of adequate knowledge and favorable attitude

Of 352 participating dentists, 176 (50.0%) met the prespecified criteria for adequate knowledge of MIH and 262 (74.4%) for a favorable attitude towards its clinical management. These binary outcomes were entered into multivariable logistic regression models to identify independent predictors after adjustment for potential confounders (Table 5). The model for adequate knowledge identified one statistically significant predictor: professionals with 6–10 years of experience showed significantly higher odds of adequate knowledge compared to those with five years or less (adjusted OR = 2.23, 95% CI: 1.14–4.37, *p* = 0.019). Although numerical trends suggested positive associations for other variables, none reached statistical significance. The model showed adequate goodness of fit (Hosmer-Lemeshow test: χ² = 6.13, df = 8, p = 0.633), with a Nagelkerke pseudo-R² of 0.072 and an AUC of 0.634, indicating acceptable but modest discriminative ability.

**Table 5:** Multivariate logistic regression model for predictors of adequate knowledge and favorable attitude towards the management of MIH.

Predictor variable	Adequate knowledge about MIH^a^						Favorable attitude towards the management of MIH^b^					
	OR Crude (95% CI)		*P*-Value	Adjusted OR^c^ (95% CI)		*P*-Value	OR Crude (95% CI)		*P*-Value	Adjusted OR^c^ (95% CI)		*P*-value
Dental Specialty												
General Dentistry		Ref.			Ref.			Ref.			Ref.	
Pediatric dentistry	1.10	(0.50–2.43)	0.810	2.38	(0.43–13.09)	0.319	0.64	(0.27–1.50)	0.305	0.76	(0.13–4.38)	0.758
Orthodontics	0.95	(0.41–2.22)	0.914	2.12	(0.38–11.72)	0.388	0.61	(0.24–1.50)	0.279	0.67	(0.12–3.77)	0.647
Endodontics	0.87	(0.37–2.07)	0.761	1.94	(0.34–11.07)	0.458	0.86	(0.32–2.29)	0.761	0.91	(0.15–5.58)	0.916
Oral Rehabilitation	1.31	(0.51–3.39)	0.574	2.88	(0.46–18.02)	0.258	2.58	(0.58–11.53)	0.216	3.15	(0.36–27.53)	0.299
Dental Aesthetics	0.68	(0.21–2.22)	0.524	1.45	(0.21–10.16)	0.707	0.42	(0.13–1.40)	0.158	0.57	(0.08–4.11)	0.580
Other specialties	0.60	(0.28–1.30)	0.198	1.10	(0.21–5.82)	0.910	0.64	(0.28–1.44)	0.278	0.74	(0.13–4.09)	0.729
Professional experience												
≤5 years		Ref.			Ref.			Ref.			Ref.	
6–10 years	1.84	(1.07–3.15)	0.028	2.23	(1.14–4.37)	0.019	0.59	(0.32–1.08)	0.087	0.66	(0.31–1.38)	0.268
11–15 years	0.55	(0.26–1.16)	0.115	0.76	(0.30–1.89)	0.555	0.42	(0.20–0.89)	0.024	0.45	(0.17–1.17)	0.102
>15 years	1.05	(0.61–1.81)	0.867	1.39	(0.51–3.77)	0.523	0.90	(0.46–1.74)	0.751	0.72	(0.23–2.22)	0.567
Academic level												
Third level		Ref.			Ref.			Ref.			Ref.	
Specialization	0.79	(0.49–1.27)	0.327	0.43	(0.08–2.21)	0.312	0.71	(0.42–1.23)	0.222	1.01	(0.19–5.34)	0.995
Master	0.87	(0.43–1.79)	0.714	0.57	(0.12–2.62)	0.468	0.65	(0.30–1.43)	0.289	0.83	(0.17–3.95)	0.811
PhD	1.11	(0.33–3.75)	0.865	0.74	(0.11–5.24)	0.766	1.35	(0.28–6.46)	0.707	1.84	(0.19–17.69)	0.596
Work sector												
Private		Ref.			Ref.			Ref.			Ref.	
Public	1.33	(0.75–2.37)	0.332	1.44	(0.76–2.72)	0.258	1.30	(0.65–2.60)	0.465	1.40	(0.66–2.97)	0.376
Both sectors	0.95	(0.49–1.82)	0.867	1.22	(0.59–2.53)	0.596	0.77	(0.38–1.57)	0.475	0.90	(0.40–2.01)	0.795
Age Groups												
≤30 years		Ref.			Ref.			Ref.			Ref.	
31–40 years	1.03	(0.63–1.69)	0.900	0.83	(0.43–1.57)	0.562	0.51	(0.29–0.92)	0.024	0.74	(0.35–1.57)	0.435
41–50 years	0.76	(0.38–1.55)	0.455	0.66	(0.23–1.91)	0.448	0.81	(0.35–1.88)	0.619	1.24	(0.37–4.13)	0.726
>50 years	0.89	(0.45–1.75)	0.730	0.62	(0.20–1.93)	0.406	0.93	(0.40–2.15)	0.863	1.02	(0.28–3.79)	0.974
Sex												
Male		Ref.			Ref.			Ref.			Ref.	
Female	0.74	(0.47–1.17)	0.201	0.69	(0.42–1.14)	0.146	0.70	(0.40–1.21)	0.197	0.83	(0.46–1.52)	0.551
Previous information about MIH												
No		Ref.			Ref.			Ref.			Ref.	
Yes	1.15	(0.75–1.77)	0.514	1.24	(0.77–2.00)	0.379	0.73	(0.45–1.18)	0.195	0.73	(0.42–1.26)	0.256

a Model fit—Adequate knowledge about MIH: Hosmer-Lemeshow *χ*^2^ = 6.13, df = 8, *p* = 0.633; Nagelkerke R^2^ = 0.072; AUC=0.634.

b Model fit—Favorable attitude towards the management of MIH: Hosmer-Lemeshow *χ*^2^ = 5.74, df = 8, *p* = 0.676; Nagelkerke R^2^ = 0.076; AUC=0.642.

c Models adjusted for dental specialty, professional experience, academic level, work sector, age group, sex, and previous information on MIH.

The model for favorable attitude did not identify statistically significant independent predictors after multivariate adjustment (all *p* > 0.05). A trend towards reduced odds was observed for professionals with 11–15 years of experience (adjusted OR = 0.45, 95% CI: 0.17–1.17, *p* = 0.102) and for those working in both sectors (adjusted OR = 0.90, 95% CI: 0.40–2.01, *p* = 0.795). Previous information on MIH did not emerge as a significant predictor of favorable attitude (adjusted OR = 0.73, 95% CI: 0.42–1.26, *p* = 0.256). The model demonstrated adequate goodness of fit (Hosmer-Lemeshow test: χ² = 5.74, df = 8, p = 0.676), with a Nagelkerke pseudo-R² of 0.076 and an AUC of 0.642.

The comparison between crude and adjusted odds ratios revealed minimal changes in the magnitude of effects, suggesting limited confounding between the predictor variables evaluated. Both models showed low explanatory power (Nagelkerke R^2^ < 0.08), indicating that unmeasured factors contribute substantially to the variability in knowledge and attitudes towards MIH.

## 4 Discussion

This study provides evidence on the perception, knowledge, and attitudes of dentists towards MIH in Ecuador. The findings show a professional community that recognizes MIH as a prevalent and growing clinical challenge yet faces significant gaps in formal training and standardized management approaches. The sociodemographic profile of the sample, which is made up predominantly of young, female practitioners based in urban areas and working in private general practice, reflects broader trends in the feminization and urbanization of dentistry that have been documented globally (3, 4, 9, 13–17). However, this profile raises a critical concern specific to Ecuador: the concentration of professionals in urban areas means that rural populations are not receiving adequate dental care at a time when reports of MIH are increasing. This finding highlights the need for health workforce policies that incentivize rural practice and integrate MIH training into undergraduate curricula before geographical specialization occurs.

The high frequency of MIH observed by participants and the widespread perception of an increasing incidence are consistent with previous studies conducted in Spain (14), Portugal (3), Syria (15), Greece (9), Norway (16), Hong Kong (13) and Switzerland (17). However, these perceptions should be interpreted with caution as they may reflect a genuine epidemiological increase, greater diagnostic awareness due to recent attention to MIH in the literature, or a combination of both. The significant variation in observation frequency across specialties likely reflects differential patient populations and referral patterns rather than true prevalence differences. Pediatric dentists, who treat the age group most affected by MIH, reported the highest weekly observation rates (25).

Regarding clinical knowledge, the near-universal identification of demarcated opacities as the predominant manifestation is encouraging and consistent with other populations (3, 14, 17). Nevertheless, this finding should be interpreted with caution, as demarcated opacities can be confused with dental fluorosis (25, 26) and dental caries (21, 22), both highly prevalent conditions in Ecuador. The absence of formal training in differential diagnosis, as evidenced by reliance on the internet as the primary source of information across all specialties (9), raises the possibility that the reported recognition rates may overestimate actual diagnostic accuracy. This concern is reinforced by recent evidence showing that dental students across different educational systems demonstrate significant knowledge gaps regarding MIH identification and management (19, 25, 26). Future studies incorporating clinical vignettes with differential diagnoses or calibrated clinical examinations would provide more robust evidence of diagnostic competence (27).

The predominance of antibiotics as the perceived main etiological factor aligns with findings from Syria (15) but contrasts with populations where genetic factors were prioritized (9). This diversity across countries is not surprising given the genuinely multifactorial and still incompletely understood etiology of MIH (5, 13). Notably, the single-response format of the questionnaire likely constrained the expression of this complexity, as clinicians may simultaneously recognize multiple contributing factors. The attribution of MIH primarily to antibiotics has practical implications for patient counseling: dentists may inadvertently overemphasize pharmaceutical causes while underrepresenting environmental, nutritional, and genetic contributions, potentially generating unnecessary parental anxiety about medication use (28).

The perception of MIH management as challenging, reported by most participants, is a universal finding across all studied populations (3, 9, 13–17, 25). The identification of restoration longevity as the primary concern across all specialties reflects the well-documented clinical reality that hypomineralized enamel compromises adhesive bonding, leading to higher failure rates particularly with resinous materials requiring strict isolation protocols (4, 29, 30). The preference for glass ionomer over composite resin is consistent with Portuguese (3) and Syrian (15) dentists, and may represent a pragmatic clinical response: glass ionomer offers moisture tolerance, fluoride release, and biocompatibility (29) that are advantageous in the challenging clinical conditions posed by MIH, despite inferior mechanical and aesthetic properties (31, 32). This therapeutic pattern contrasts with Swiss dental students who prioritized aesthetics and durability (17), a difference that may reflect not only training background but also patient expectations and healthcare system resources. The therapeutic variability observed across both clinical cases, with no significant differences between specialties, underscores the absence of locally adapted clinical protocols and highlights the urgent need for evidence-based decision trees for MIH management in the Ecuadorian context.

One of the strengths of the study was the fact that it had a sample that included participants from 22 of Ecuador's 24 provinces, which supports the reliability of the results, even considering that the highest percentage of participants belonged to Pichincha, where the largest number of dental training institutions practice. In the same way, the use of a previously validated questionnaire (14) that was open to the participants for a limited time allowed its control, making it a reliable instrument.

On the other hand, this study has several limitations that should be considered when interpreting the findings. Convenience recruitment from attendees at five FOE professional meetings may have introduced selection and volunteer bias, potentially overrepresenting professionals with greater interest in MIH. Uneven participation across specialties also left some groups underrepresented, limiting the precision of between-specialty comparisons. In addition, the absence of comparable studies from Ecuador or similar settings limits contextual interpretation. Despite these limitations, the inclusion of participants from 22 of the 24 provinces of Ecuador and the use of a previously validated instrument strengthen the reliability of the findings. The results should be considered a starting point for future studies with probabilistic sampling designs and larger subgroup sizes, constituting a basis for restructuring curricula in dental training institutions and developing continuous education policies on enamel developmental defects.

## 5 Conclusions

MIH was perceived by Ecuadorian dentists as a clinically important and increasingly frequent condition that remains difficult to diagnose and manage. Despite generally acceptable knowledge of its clinical features, relevant diagnostic and therapeutic gaps remain. Professional experience of 6–10 years emerged as the only significant predictor of adequate knowledge, while no independent predictors of favorable clinical attitudes were identified, suggesting that multiple unmeasured factors influence professional confidence. These findings underscore the need for the integration of formal education strategies on MIH across all career stages, the development of standardized clinical protocols, and the strengthening of national research on enamel developmental defects.

## Data Availability

The raw data supporting the conclusions of this article will be made available by the authors, without undue reservation.
